# Amelioration of pregnancy-associated osteoporosis after treatment with vitamin K_2_: a report of four patients

**DOI:** 10.3109/03009734.2012.676573

**Published:** 2012-08

**Authors:** Hiroyuki Tsuchie, Naohisa Miyakoshi, Michio Hongo, Yuji Kasukawa, Yoshinori Ishikawa, Yoichi Shimada

**Affiliations:** Department of Orthopedic Surgery, Akita University Graduate School of Medicine, 1-1-1 Hondo, Akita 010-8543, Japan

**Keywords:** Pregnancy-associated osteoporosis, vertebral fracture, vitamin K2

## Abstract

We demonstrate for the first time therapeutic effects of vitamin K_2_ (menatetrenone) on pregnancy-associated osteoporosis with multiple vertebral fractures in four cases. Due to its safety, vitamin K_2_ presents itself as a treatment option for women with pregnancy-associated osteoporosis. Desirably, future controlled studies should verify these findings.

## Introduction

Pregnancy-associated osteoporosis is a rare disease which causes vertebral compression fractures from the late gestatory period to several months after delivery. Although stopping breast-feeding combined with calcium intake are common treatments for this disease, there has been no report using vitamin K_2_ (menatetrenone), which is one therapeutic option for osteoporosis. We herein describe four cases showing a favorable course when prescribed vitamin K_2_.

## Patients and methods

The ages of the four patients ranged from 30 to 34 years, with a mean of 31.5 years. All four patients suffered from severe back pain, and plain radiographs and magnetic resonance imaging (MRI) showed multiple vertebral fractures related to their normal pregnancies.

Bone mineral density (BMD) of the lumbar spine (L2–4) and proximal femur was measured by dual-energy X-ray absorptiometry before or during the treatment. On laboratory examinations with conventional techniques, cross-linked N-telopeptide of type I collagen (NTX) in urine or serum, bone-specific alkaline phosphatase (BAP), serum intact parathyroid hormone (PTH), serum calcium (Ca), and/or inorganic phosphate (IP) were measured at the beginning or during treatment with menatetrenone.

## Case reports (Table I)

### Case 1

A lactating 30-year-old woman developed back pain 4 weeks after her first delivery following a normal pregnancy. She went to a local clinic for treatment, but the pain did not lessen, and she was transferred to our out-patient clinic with back pain for 3 months. She was previously healthy and did not take any medicine such as corticosteroids or thyroid hormones. Physical examinations demonstrated only tenderness of the back. Vertebral fractures of the 8th, 10th, and 12th thoracic (T) and 1st lumbar (L) vertebrae were seen on plain radiographs and MRI. BMD of the lumbar spine (L2–4: 0.714 g/cm^2^, T-score: –2.9 SD) and proximal femur (0.589 g/cm^2^, T-score: –3.8 SD) confirmed the presence of associated osteoporosis. On laboratory examinations, NTX in urine was high (151.4 nmolBCE/L, normal range 9.3–54.3 nmolBCE/L). However, other laboratory examinations including serum BAP, serum PTH, and serum Ca were within normal ranges. Because she was instructed to wear a corset by a previous doctor, we advised against breast-feeding and prescribed a daily intake of 45 mg of vitamin K_2_ and 1,200 mg of calcium carbonate. One year after the initial consultation, her back pain had completely disappeared, and she did not have any recurrence. NTX in urine improved (14.0 nmolBCE/L). BMD at 2 years after the initial visit showed improvement (L2–4: 0.749 g/cm^2^, T-score: –2.4 SD; and proximal femur: 0.674 g/cm^2^, T-score: –1.7 SD).

**Table I.** Clinical data of the four cases.

			Case 3		
	Case 1	Case 2	1st delivery	2nd delivery	Case 4
Age, y	30	31	23	31	34
Onset (week)	4	2	8	–	1
Delivery	1st	1st	1st	2nd	1st
Beginning of treatment (M)	3	3	14	–	3
Compression fracture	T8,10,12; L1	T8,9,12; L1,4	T7,12; L1	–	T6,8,12
BMD lumbar spine (g/cm^2^)					
Before treatment	0.71 (3M)	0.70 (3M)	0.75 (14M)	0.79 (–11M)	0.90 (3M)
After treatment	0.75 (24M)	N.A.	0.78 (25M)	0.78 (6M)	0.92 (12M)
BMD femur (g/cm^2^)					
Before treatment	0.59 (3M)	0.59 (3M)	0.54 (14M)	0.57 (–11M)	0.75 (3M)
After treatment	0.67 (24M)	N.A.	0.53 (25M)	0.56 (6M)	0.78 (12M)
NTX (nmolBCE/L)					
Before treatment	151 (3M)^a^	25 (3M)	52.2 (13M)^a^	N.A.	31 (1M)
After treatment	14 (12M)^a^	18 (10M)	9.3 (24M)^a^	N.A.	9 (8M)
BAP (U/L)					
Before treatment	23 (3M)	46 (3M)	N.A.	N.A.	32 (1M)
After treatment	14 (24M)	13 (10M)	18.5 (24M)	N.A.	13 (8M)
Ca (mg/dL)					
Before treatment	9.4 (3M)	9.5 (3M)	9.2 (13M)	N.A.	10.9 (1M)
After treatment	9.2 (24M)	9.2 (9M)	8.8 (24M)	N.A.	9.4 (6M)
IP (mg/dL)					
Before treatment	4.4 (3M)	3.7 (3M)	3.7 (13M)	N.A.	4.6 (1M)
After treatment	2.8 (24M)	3.9 (9M)	3.4 (24M)	N.A.	3.4 (6M)
Intact PTH (pg/L)					
Before treatment	61 (3M)	N.A.	22 (13M)	N.A.	7 (1M)
After treatment	13 (24M)	N.A.	N.A.	N.A.	28 (4M)

^a^NTX in urine. M = month after delivery.

### Case 2

**Figure 1.**
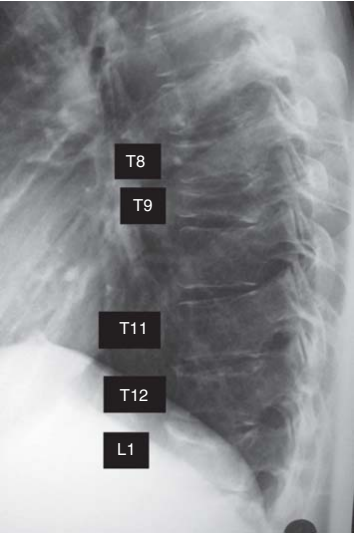
Lateral radiograph of the vertebrae in case 2. Radiograph showed mild compression changes at the 8th, 9th, and 12th thoracic vertebrae and 1st lumbar vertebra.

**Figure 2.**
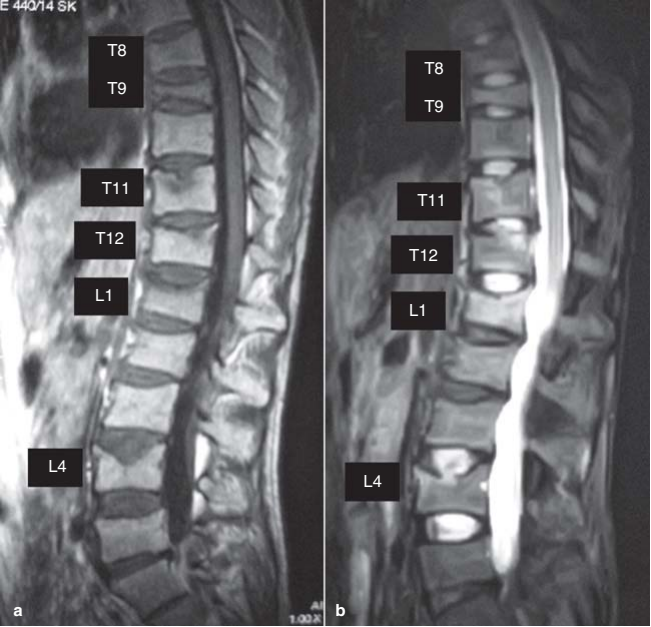
Magnetic resonance imaging (MRI) of the vertebrae in case 2. MRI showed low-signal intensities on T1-weighted images (A) and high intensities on T2-weighted images (B) at the 8th, 9th, 11th, and 12th thoracic vertebrae and 1st and 4th lumbar vertebrae.

A 31-year-old woman with back pain was admitted 3 months after her first delivery. Her back pain had started 2 weeks after delivery, and she went to a local clinic for treatment. However, the pain did not lessen, and she was transferred to our out-patient clinic. She had a past history of eating disorder and left femoral neck fracture 2 years before. Physical examinations demonstrated only tenderness of her back. Vertebral fractures of the T8, 9, 11, and 12, and L1 and 4 vertebrae were seen on plain radiographs and MRI (Figures 1 and 2). BMD of the lumbar spine (L2–4: 0.698 g/cm^2^, T-score: –2.8 SD) and proximal femur (0.585 g/cm^2^, T-score: –2.3 SD) confirmed the presence of associated osteoporosis. Serum NTX and BAP were 25 nmolBCE/L (normal range 7.5–16.5 nmolBCE/L) and 46 U/L (normal range 9.6–35.4 U/L), respectively. There was no abnormal value on laboratory examinations of other parameters. Breast-feeding had been advised against 1 month before, so we instructed her to wear a corset and prescribed a daily intake of 45 mg of vitamin K_2_. Six months after the initial consultation, her back pain had decreased significantly, and she did not experience any recurrence. The bone metabolic markers both of serum NTX and BAP were suppressed to 18 nmolBCE/L and 13 U/L, respectively, after 10 months of treatment.

### Case 3

A 31-year-old woman in her second pregnancy was transferred to our out-patient clinic due to pregnancy-associated osteoporosis. She developed back pain 2 months after her first delivery following a normal pregnancy 7 years earlier. She went to a local clinic for treatment, and compression fractures of the T7, 12, and L1 vertebrae were seen on plain radiographs and MRI. She was previously healthy and did not take any medicine. There were no abnormal values on laboratory examinations. She was instructed to wear a corset only, and not to continue breast-feeding. Because BMD showed the presence of associated osteoporosis (L2–4: 0.750 g/cm^2^, T-score: –2.66 SD; and proximal femur: 0.539 g/cm^2^, T-score: –4.55 SD), she was transferred to our out-patient clinic. We prescribed a daily intake of 45 mg of vitamin K_2_, and her back pain completely disappeared. BMD at 2 years and 1 month after first pregnancy still showed associated osteoporosis (L2–4: 0.775 g/cm^2^, T-score: –2.46 SD; and proximal femur: 0.569 g/cm^2^, T-score: –4.43 SD), and she continued to take vitamin K_2_ for osteoporosis treatment at her own request.

We advised against breast-feeding on her second pregnancy and prescribed a daily intake of 45 mg of vitamin K_2_ 2 weeks after delivery. We continued to prescribe vitamin K_2_ until 6 months after delivery, and no back pain appeared. BMD at 6 months after delivery showed no change (L2–4: 0.778 g/cm^2^, T-score: –2.1 SD; and proximal femur: 0.564 g/cm^2^, T-score: –2.7 SD).

### Case 4

**Figure 3.**
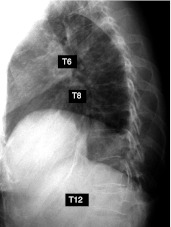
Lateral radiograph of the vertebrae in case 4. Radiograph showed mild compression changes at the 6th, 8th, and 12th thoracic vertebrae.

**Figure 4.**
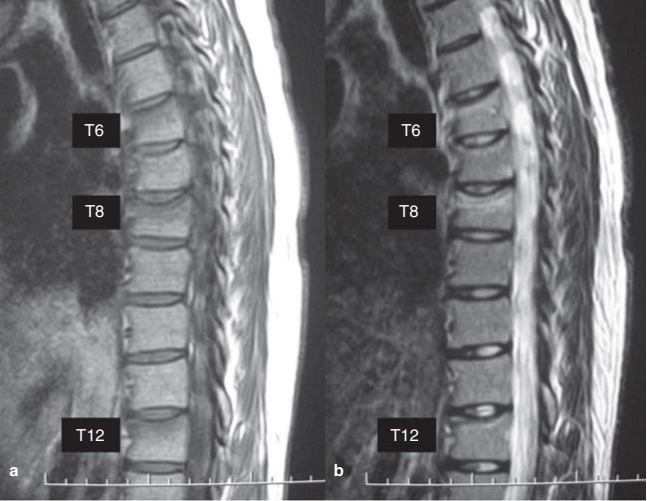
Magnetic resonance imaging (MRI) of the vertebrae in case 4. MRI showed low signal intensities on T1-weighted images (A) and high intensities on T2-weighted images (B) at the 6th, 8th, and 12th thoracic vertebrae.

A 34-year-old woman developed back pain 1 week after her first delivery following a normal pregnancy. She went to a local clinic for treatment, and compression fractures of the T6, 8, and 12 vertebrae were seen on plain radiographs and MRI (Figures 3 and 4). BMD showed a mild decline (L2–4: 0.901 g/cm^2^, T-score: –1.0 SD; and proximal femur: 0.749 g/cm^2^, T-score: –1.0 SD). On laboratory examinations, serum NTX (30.8 nmolBCE/L) and serum Ca (10.9 mg/dL, normal range 7.8–10.1 mg/dL) were elevated, and serum intact PTH showed a decrease (7 pg/mL, normal range 10–65 pg/mL). However, other laboratory examinations including serum BAP were within the normal ranges. She was instructed to wear a corset and avoid breast-feeding, and prescribed a daily intake of non-steroidal anti-inflammatory drugs (NSAIDs). However, her back pain did not lessen, and compression changes of the thoracic vertebrae worsened on plain radiographs. So she was transferred to our out-patient clinic with back pain for 3 months.

**Figure 5.**
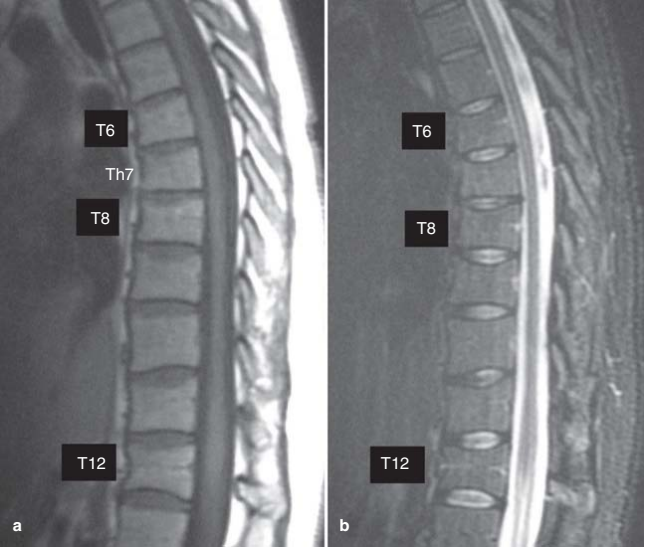
Magnetic resonance imaging (MRI) of the vertebrae in case 4 at 7 months after prescribing vitamin K_2_. MRI showed no signal intensities on T1- (A) and T2- (B) weighted images.

Physical examinations demonstrated only tenderness of her back. She was previously healthy without any other disease. We prescribed a daily intake of 45 mg of vitamin K_2_, and her back pain decreased gradually. At 5 months after prescribing vitamin K_2_, her back pain had completely disappeared. MRI after 7 months of treatment showed no signal intensities on T1- and T2-weighted images (Figure 5). BMD after 12 months of treatment was 0.92 g/cm^2^ (T-score: –0.8 SD) of L2–4 and 0.78 g/cm^2^ (T-score: –0.7 SD) of the proximal femur. Although we finished prescribing vitamin K_2_ at this time, she did not experience any recurrence.

## Discussion

Pregnancy-associated osteoporosis, which was first described as a clinical syndrome by Nordin and Roper, is a relatively rare disease in young women (1). Although many cases of pregnancy-associated osteoporosis have been reported, the etiology of this condition is unknown. There are several hypotheses regarding causes of pregnancy-associated osteoporosis: 1) increase of parathyroid hormone-related peptide (PTH-rP) secretion by lactation, 2) increase of calcium supply to fetal bone and breast-milk, 3) decrease of estrogen after delivery, 4) osteopenia existing before pregnancy, and 5) heritable factors (2–6).

For the treatment of pregnancy-associated osteoporosis, most reports have dealt with conservative treatment such as weaning, bed rest, corset application, and medical treatment which includes calcium, vitamin D, bromocriptine, and analgesics (7,8).

Vitamin K_2_ is one option for osteoporosis treatment. The effects of vitamin K_2_ on bone are improving the bone quality by promoting γ-carboxylation of osteocalcin, and advancing bone formation and calcification through steroid and xenobiotic receptors (SXR) (9,10). One report mentions that vitamin K_2_ effectively prevents fractures and sustains bone mineral density in osteoporosis (11). All our presented cases showed a favorable course following treatment with vitamin K_2_.

There are some reports of good results employing bisphosphonate and operative treatment (7,12). Bisphosphonate increases bone mineral density but, its safety in women who are possibly pregnant has not been established because of its accumulation in bone (13). Although surgical treatment reduces symptoms early, we cannot completely ignore the risk of surgery. Vitamin K_2_ is administered to newborns or pregnant women for the treatment of melena neonatorum (14), so this medicine is safer than these other treatments. It may be difficult to conclude that vitamin K_2_ treatment improved symptoms of post-pregnancy osteoporosis earlier than these treatments. However, we can easily use it for women who may be pregnant due to its safety.

In conclusion, we present four pregnancy-associated osteoporosis cases showing a favorable course on treatment with vitamin K_2_. There is to our knowledge, no previous report on the use of vitamin K_2_ use for pregnancy-associated osteoporosis treatment. Although stopping lactation is the basis of treatment, we should consider vitamin K_2_ as a choice of subsidiary treatment, because of its promising effects and safety.
